# Evaluation of long-term conservation agriculture and crop intensification in rice-wheat rotation of Indo-Gangetic Plains of South Asia: Carbon dynamics and productivity

**DOI:** 10.1016/j.eja.2017.08.006

**Published:** 2017-10

**Authors:** S.K. Samal, K.K. Rao, S.P. Poonia, Rakesh Kumar, J.S. Mishra, Ved Prakash, S. Mondal, S.K. Dwivedi, B.P. Bhatt, Sushanta Kumar Naik, Anup Kumar Choubey, V. Kumar, R.K. Malik, Andrew Mc Donald

**Affiliations:** a ICAR Research Complex for Eastern Region, P.O. Bihar veterinary College, Patna -800 014, Bihar, India; b Cereal Systems Initiative for South Asia, Research Platform, ICAR Research Complex for Eastern Region, Patna -800 014, Bihar, India; c ICAR Research Complex for Eastern Region, Research Centre, Ranchi -834010, Plandu, Jharkhand, India; d International Rice Research Institute, Los Banos, Philippines; e Cereal Systems Initiative for South Asia, Kathmandu, Nepal

**Keywords:** IGP, Indo Gangetic plains, RWCS, rice wheat cropping system, CA, conservation agriculture, S1, scenario 1, S2, scenario 2, S3, scenario 1, S4, scenario 4, OC, organic carbon, SOC, soil organic carbon, C stock, carbon stock, TOC, total organic carbon, CMI, carbon management index, NT, no tillage, CT, conventional tillage, MBC, microbial biomass carbon, DHA, dehydrogenase activity, FDA, flouroscein di acetate activity, AlkP, alkaline phosphatase activity, Carbon stock, Carbon fractions, Carbon budgeting, Crop yield, Conservation agriculture

## Abstract

In the context of deteriorating soil health, stagnation of yield in rice-wheat cropping system (RWCS) across Indo- Gangetic plains (IGP) and environmental pollution, a long term field experiment was conducted during 2009–2016 taking four crop scenarios with conservation agriculture (CA), crop intensification and diversified cropping as intervening technology aiming to evaluate the sustainability of the systems. Scenario 1 (S1) represented conventional farmers’ practice of growing rice and wheat with summer fallow. In scenario 2 (S2) and scenario 3 (S3), legume crop was taken along with rice and wheat with partial CA and full CA, respectively. Conventional RWCS was replaced with rice-potato + maize- cowpea cropping system with partial CA in scenario 4 (S4). The S3 scenario registered highest total organic carbon (TOC) stock of 47.71 Mg C ha^−1^ and resulted in significant increase of 14.57% over S1 (Farmer’s practice) in 0–30 cm soil depth after 7 years of field trial. The S4 scenario having intensified cropping systems recorded lowest TOC of 39.33 Mg C ha^−1^ and resulted in significant depletion of 17.56% in C stock with respect to S3 in 0–30 cm soil depth. The TOC enrichment was higher in S2, S3 and S4 scenario in the surface soil (0–10 cm) compared to S1. At lower depth (20–30 cm), the TOC enrichment was significantly higher in S2 (12.82 Mg C ha^−1^) and S3 (13.10 Mg C ha^−1^ soil) over S1 scenario. The S2 and S3 scenario recorded highest increased allocation of TOC (3.55 and 6.13 Mg C ha^−1^) to passive pool over S1. The S2 (15.72 t ha^−1^), S3 (16.08 t ha^−1^) and S4 (16.39 t ha^−1^) scenarios recorded significantly higher system rice equivalent yield over S1 (10.30 t ha^−1^). Among the scenarios, S3 scenario had greater amount of total soil organic carbon, passive pool of carbon and higher system rice equivalent yield, thus, is considered the best cropping management practice to maintain soil health and food security in the middle IGP.

## Highlights

• Conservation agriculture (CA) practice meaningfully stabilized organic carbon (OC) upto lower depth of soil.
• Crop intensification with nutrient exhaustive crops lowers OC stabilization in soil even after adoption of CA practice.
• Farmers practice of growing rice and wheat produce less yield with less storage of carbon in soil.
• Rice-wheat-legume crop with CA proved as most efficient in terms of crop yield, soil health and carbon sequestration.

## 1 Introduction

Rice-wheat is the major production system covering an area of 13.5 million hectares across the Indo-Gangetic Plains (IGP) of south Asia (Ladha et al., 2003) and feeds about 1/5th of world population (Saharawat et al., 2010). But after impressive gain in production due to various inputs used and adoption of improved agronomic practices during green revolution, now the sustainability of the system is questionable. Conventionally grown rice and wheat are highly money, water and energy intensive. Conventional rice requires puddling and seed bed preparation, which needs more water and labour; and in turn breaks soil aggregates exposing the soil for oxidation of organic carbon (Mondal et al., 2016). Although puddling has its advantage in terms of better weed control, lesser percolation loss and providing anaerobic condition to rice, it leads to delayed sowing of wheat, which requires well drained soil with good tilth. Kumar et al. (2008) reported 8% yield reduction in wheat yield when grown after puddled transplanted rice in comparison to wheat grown after direct seeded rice under unpuddled condition. Conventionally grown rice-wheat leads to depletion of SOC at the rate 0.13 t ha^−1^ yr^−1^ from 0 to 0.6 m depth of eastern IGP (Sapkota et al., 2017). Declining soil health, decreasing water use efficiency and environmental pollution are major sustainability issues of RWCS (Bhatt et al., 2016). Sequestering soil organic carbon (SOC) is the key strategy to improve soil health and mitigating climate change. Furthermore, increased allocation of SOC into passive pools of longer residence time helps to achieve higher carbon sequestration in soils (Mandal et al., 2008). Pragmatic solution of the aforesaid concerns is conservation Agriculture (CA), which includes practices like reduced tillage (or no tillage), residue incorporation and crop rotation. These practices are needed to be adopted by integrating into a set of appropriate management condition for reversing loss of soil organic carbon (SOC).

In the beginning no tillage (NT) practice was aimed to conserve soil, moisture, to reduce cost of production (Holland, 2004). Beyond this, the practice has multiple benefits in increasing the overall system performance. Jat et al. (2014) reported significantly higher rice-wheat system grain yield in conservation tillage as compared to conventional tillage (CT) practice in IGP of India. Carbon is the central element that determines soil fertility through mediating the release of various plant available nutrients in soil, thus determines yield of crops. Simultaneously, it improves the soil resilience through buffering various soil properties, which provide good soil environment for plant growth (Chan, 2010). In addition to this, higher SOC improves microbial activity and better physical environment in soil, thus ensures better health of soil. Number of studies has reported increase in carbon content in soil under NT to CT. However some other studies showed that NT can only accumulate carbon in surface soil upto few centimeters of depth (0–20 cm) and that increase in carbon is counteracted by depletion of carbon in lower depths (20–25 cm) (Dolan et al., 2006). When whole soil profile was taken into account NT didn’t accumulate additional amount of carbon as compared to CT, except in the upper soil layer (0–10 cm) and only there was change in SOC distribution in different layers of soil in NT in comparison to CT (Luo et al., 2010, Novak et al., 2009). Some researchers found that the increase in organic carbon in soil under NT is not too large as expected compared to CT (Virto et al., 2012). The role of NT in increasing carbon in soil has been questioned in recent studies (Ogle et al., 2012). This is an important researchable issue, which needs to be assessed thoroughly for identifying stable SOC sink upto lower depth of soil along with system performance.

Evidences from research suggest that inclusion of legume in cereal–cereal rotation enhance soil quality and raises organic carbon level in soil (Ghosh et al., 2012). It greatly enhances SOC status of soil when adopted along with CA practice (Lal, 2004). Growing cover crop like summer mung bean (*Vigna radiata*) during intervening period (period from wheat harvesting to sowing/transplanting of rice) has tremendous capacity to improve land and water productivity through in-situ soil moisture conservation (Bhatt et al., 2016). Researchers can therefore, think of increasing SOC level in soil though crop intensification, by adding legume crop in RWCS or through some alternative diversified cropping system, which will provide economic benefits to farmers. But there coexists two contrasting concepts in this regard. One postulates that crop intensification result into addition of more biomass carbon, thus, enhances SOC. Another suggests that intensification leads to depletion of organic carbon through returning only fraction of organic carbon fixed by photosynthesis. In addition to this, the added residue facilitates the microbial decay of organic matter resulting into degradation of soil aggregates (Janzen, 2006). A number of researches have been carried out by including a legume crop viz. mung bean in RWCS under CA practice to evaluate its yield benefit and SOC. But, the studies on the subject are very scarce which evaluated some alternative diversified cropping system in terms of yield and SOC status.

Total organic carbon (TOC), which is composed of labile and recalcitrant fraction, cannot give sufficient information about mechanism of carbon accumulation and loss. Labile carbon fractions are sensitive to small changes in SOC (Xia et al., 2010) and greatly influence microbial transformation process in soil (Haubensak et al., 2002). The relative proportion of the aforesaid fractions determines the quality of soil and forms the basis for study of carbon dynamics. Under CA practices carbon dynamics study is highly dependent on soil type and climate. Long-term experiment forms the basis of assessment of long-term changes in SOC and sustainability of agricultural production systems (Ladha et al., 2003). It is also argued that perceiving the overall process is more important in tropical and subtropical condition in general and in IGP in particular, because these regions are inherently low in organic carbon and production system is highly vulnerable (Mandal et al., 2007, Patil et al., 2014).

Keeping these facts in view, in the present investigation was undertaken to evaluate the four different cropping scenarios of CA and crop intensification practices including farmer’s practice of growing rice-wheat, rice-wheat-mung bean and an alternative diversified cropping system for yield and carbon stabilization capacity in middle IGP. This investigation was made to address the issues of declining soil health and yield stagnation in conventional rice-wheat cropping system and we have tried to answer the following questions: (1) Is CA superior over farmer’s practice in terms of crop yield and carbon stabilization? (2) Is intensive cultivation practice with mung bean in RWCS or alternative diversified cropping system can be adopted as an alternate superior technology in terms of TOC build up and crop yield?

## 2 Materials and methods

### 2.1 Study site and experimental design

**Fig. 1:**
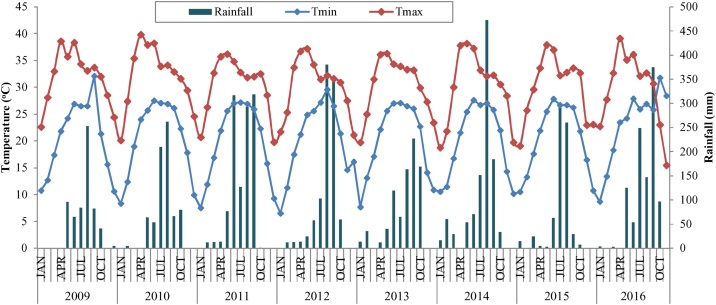
Monthly total rainfall, mean monthly maximum and minimum temperature prevailing during the experimental period.

A long-term field experiment was initiated in winter season 2009 taking four cropping system (scenarios) varying in crop rotation, tillage, establishment method, residue management, and other management laid out in a randomized complete block design in three replications at experimental farm of Indian Council of Agricultural Research (ICAR) Research Complex for Eastern Region, Patna (located between 25.5941° N, 85.1376° E and an altitude of 50 m asl) in Bihar, India. The study was carried out for a period of seven years (2009–2016). The research station falls in middle IGP of India having subtropical humid climate. During the experimental period, the distribution of rainfall over time and intensity in the rainy season was very erratic. The lowest rainfall (626 mm) was recorded in the year of 2009 and 2010 and the highest (1172 mm) in 2011with average rainfall of 902.2 mm. The lowest minimum temperature (6.5 °C) was recorded in January 2012 and the highest (39.8 °C) in April 2010. Generally, maximum temperature exceeding 35 °C was observed in month of April and May and the lowest was observed in January. Rise and fall of maximum temperature is controlled by thunderstorm activity during summer and that of minimum temperature by passage of western disturbance in winter. The average monthly temperature (Tmax and Tmin) and rainfall distribution is presented in (Fig. 1). Before start of experiment a crop of puddle transplanted rice was grown on the experimental land to homogenise the site. After harvest of the crop the land was levelled and divided into 12 plots of equal size of 0.2 ha each. The experimental soil is old alluvium with texture of surface soil was silty clay. The basic soil properties at the start of experiment including fertility status of soil and method followed for analysis are given in Table 1.

**Table 1:** Basic soil properties of experimental site before commencement of study.

Soil properties	0–15 cm		15–30 cm		Methods
	Mean ± SE	Range	Mean ± SE	Range	
Sand (%)	16.8 ± 0.3	18.4–9.6	17.5 ± 0.3	9.6–18.0	Bouyoucos (1962)
Silt (%)	41.8 ± 0.5	34.0−48.0	40.8 ± 0.5	30.0−48.0	
Clay (%)	41.4 ± 0.5	37.6–52.4	41.8 ± 0.4	38.4−2.4	
pH (1:1 soil: water	7.5 ± 0.0	7.3–7.7	7.6 ± 0.0	7.3–7.7	Jackson (1973)
EC (dS m^−1^) (1:1 soil: water)	0.33 ± 0.00	0.25−0.43	0.32 ± 0.01	0.24−0.42	
TOC (%)	0.80 ± 0.01	0.6−0.9	0.57 ± 0.01	0.45−0.84	TOC analyzer
Total N (%)	0.09 ± 0.01	0.04−0.20	0.059 ± 0.005	0.02−0.15	TN analyzer
Olsen P (mg kg^−1^)	31.73 ± 0.9	26.2–36.8	21.3 ± 1.9	10.4–31.4	Olsen et al. (1954)
Average K (mg kg^−1^)	147.8 ± 6.4	128.5–200.5	154.5 ± 4.7	138.5–192.5	Hanway and Heidel (1952)
Average Zn (mg kg^−1^)	0.83 ± 0.03	0.71−1.06	0.57 ± 0.03	0.41−0.74	Lindsay and Norvell (1978)
Average Mn (mg kg^−1^)	22.0 ± 1.1	10.1–38.3	12.2 ± 1.1	6.0−25.2	

### 2.2 Scenario description

**Fig. 2:**
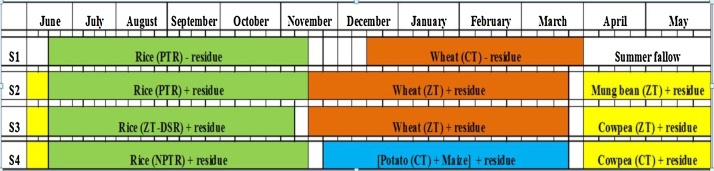
Cropping systems with their dates of sowing, harvesting and residue management practices followed in different scenarios from 2009 to 2012. PTR: puddle transplanted rice; ZT-DSR: zero till direct seeded rice; NPTR: non puddle transplanted rice; CT- conventional tillage. *Nursery seeding for transplanted rice was done on the same day as on DSR was sown. In case of transplanted rice 30 days old seedling was used for transplanting.

**Fig. 3:**
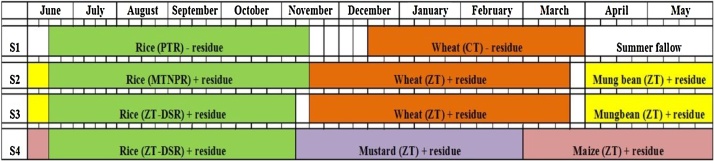
Cropping systems with their dates of sowing, residue management practices followed in different scenarios from 2013 to 2016. PTR: puddle transplanted rice; ZT-DSR: zero till direct seeded rice; MTNPR: machine transplanted non puddle rice; CT- conventional tillage. *Nursery seeding for transplanted rice was done on the same day as on DSR was sown. In case of transplanted rice 30 days old seedling was used for transplanting

Scenario 1: This scenario represents traditional farmers’ practice in which farmers take two crops in a year (rice and wheat) (Table 2). Field remains fallow during summer and wheat (*Triticum aestivum*) is sown in mid December after harvesting of long-duration rice (*Oryaza sativa*) and with ploughing of fields 3–4 times. In case of long duration transplanted rice, nursery is sown in 3rd week of June, followed by transplanting after 30 days. Subsequently, harvesting is done in last week of November. By that time soil moisture got depleted, hence, it take additional 15 days of time to get congenial soil condition for wheat sowing after ploughing of soil and giving one irrigation. This delays the wheat sowing, resulting in yield reduction. However, to maintain uniformity in different scenarios in this experiment, instead of taking long duration variety in S1, we had taken a medium duration variety (135-140 days) of rice in all the scenarios. Rice in this system is puddle transplanted. The sowing and harvesting time of all crops in different scenarios is depicted in Fig. 2, Fig. 3.

**Table 2:** Tillage, cropping systems and residue management practices followed in four cropping system scenarios.

Technological intervention	Scenario 1 (TPR-CTW)	Scenario 2 (TPR/MTNPR+R-ZTW+R-CTMB+R)	Scenario 3 (ZTDSR+R-ZTW+R-ZTC/ZTMB+R)	Scenario 4c [NPTPR/ZTDSR+R-CT(P+M)/ZTM+R-ZTC/ZTM+R]
Name of Practices followed	Farmer’s practice	Partial CA with crop intensification	Full CA with crop intensification	Partial CA with Intensified cropping system with diversification
Crop rotation	Rice-wheat- fallow	Rice-wheat-greengram	Rice-wheat-cowpea	Rice-Potato+maize-cowpea
Tillage	Rice-puddling	Rice-puddling	Rice- ZT	Rice- Unpuddled
	Wheat- CT	Wheat- ZT	Wheat- ZT	Potato+maize - CT
		Mung bean- ZT	Cowpea- ZT	Cowpea- ZT
Crop establishment method	Rice-transplanting	Rice- transplanting	Rice- DSR(drill)/TPR	Rice- transplanting
	Wheat- broadcast	Wheat- drill seeding	Wheat- drill seeding	Potato+maize- dibble
		Mung bean- drill seeding	Cowpea- drill seeding	Cowpea- relay dibble
Crop residue management	Removed	Wheat: partially (anchored) incorporated	Rice, wheat: one-third, retained on soil surface	Potato: full, incorporated
		Mung bean: full incorporated	Cowpea: full, retained on soil surface	Maize: one-third retained on soil surface
		Rice: anchored, retained on soil surface		Cowpea: full, incorporated
				Rice: one-third, incorporated

* TPR-CTW: conventional till puddled transplanted rice- conventional tille wheat; TPR/MTNPR + R-ZTW + R-CTMB + R: conventional till puddled transplanted rice/machine transplanted non puddle rice with residue- zero till wheat with residue- conventional till mung bean with residue; ZTDSR + R-ZTW + R-ZTC/ZTMB + R: zero till direct seeded rice with residue-zero till wheat with residue-zero till cowpea/zero till mung bean with residue; NPTPR/ZTDSR + R-CT(P + M)/ZTM + R-ZTC/ZTM + R: non puddle transplanted rice/zero till direct seeded rice with residue-conventional till potato and maize intercrop/zero till mustard with residue-zero till cowpea/zero till maize with residue.

Scenario 2: This scenario was outlined for increasing the system productivity and to enhance SOC status. Mungbean (*Vigna radiata*) was introduced to cover the intervening period (summer fallow covering the period from 1st week of April to 2nd week of June), which has the advantage of extra income to farmer, soil moisture conservation and addition of carbon to soil (Table 2). Wheat was timely sown (third to fourth week of November) to ensure optimum yield. Partial CA practice (soil was puddle for rice transplanting, while wheat and mung bean were sown in ZT) was adopted with less soil disturbance. On 5th year and onwards mechanical transplanting of rice was done after one dry tillage to address the issue of labour scarcity and for minimal soil disturbance (Fig. 3). It also facilitates transplanting of younger (15–20 days old) rice seedling ensuring sufficient time for timely wheat sowing. The sowing and harvesting time of all crops in different scenarios is depicted in Fig. 2, Fig. 3.

Scenario 3: This system was also designed for increasing system productivity, increasing carbon storage in soil and offsetting carbon from environment. Cowpea (*Vigna unguiculata*) was grown in intervening period. Full CA practice was implemented with no soil disturbance (Table 2). On 5th year onwards cowpea was replaced with mung bean and full biomass was retained on soil surface after harvesting of pods (Fig. 3). The reason for replacing cowpea with mung bean is its fewer market prices in comparison to mung bean and high labour requirement in its picking, which increased the production cost and ultimately incurred less profit. The sowing and harvesting time of all crops in different scenarios is depicted in Fig. 2, Fig. 3.

Scenario 4: This scenario was designed to evaluate the effect of diversified intensive cultivation on system productivity and SOC status. Wheat was replaced with potato (Solanum tuberosum) + maize (Zea mays) intercrop (Table 2). Cowpea was grown in intervening period. On 5th year onwards potato + maize intercrop and cowpea were replaced with mustard (*Brassica juncea*) and maize respectively, to lessen the fertilizer and water use (Fig. 3). Potato + maize intercrop was labour and water intensive with more soil disturbance. After normal rainfall event, it requires additional 5 number of irrigation of 6 ha cm each, hence total irrigation water required for one ha of crop is 30 ha cm. In contrast, mustard can be grown successfully in few rainfall events and with two additional irrigations of 6 ha cm each, hence total irrigation water required is 12 ha cm. It also allows one maize crop thereafter before rice. Forty percent anchored residues were kept in both maize and mustard. The sowing and harvesting time of all crops in different scenarios is depicted in Fig. 2, Fig. 3.

**Fig. 4:**
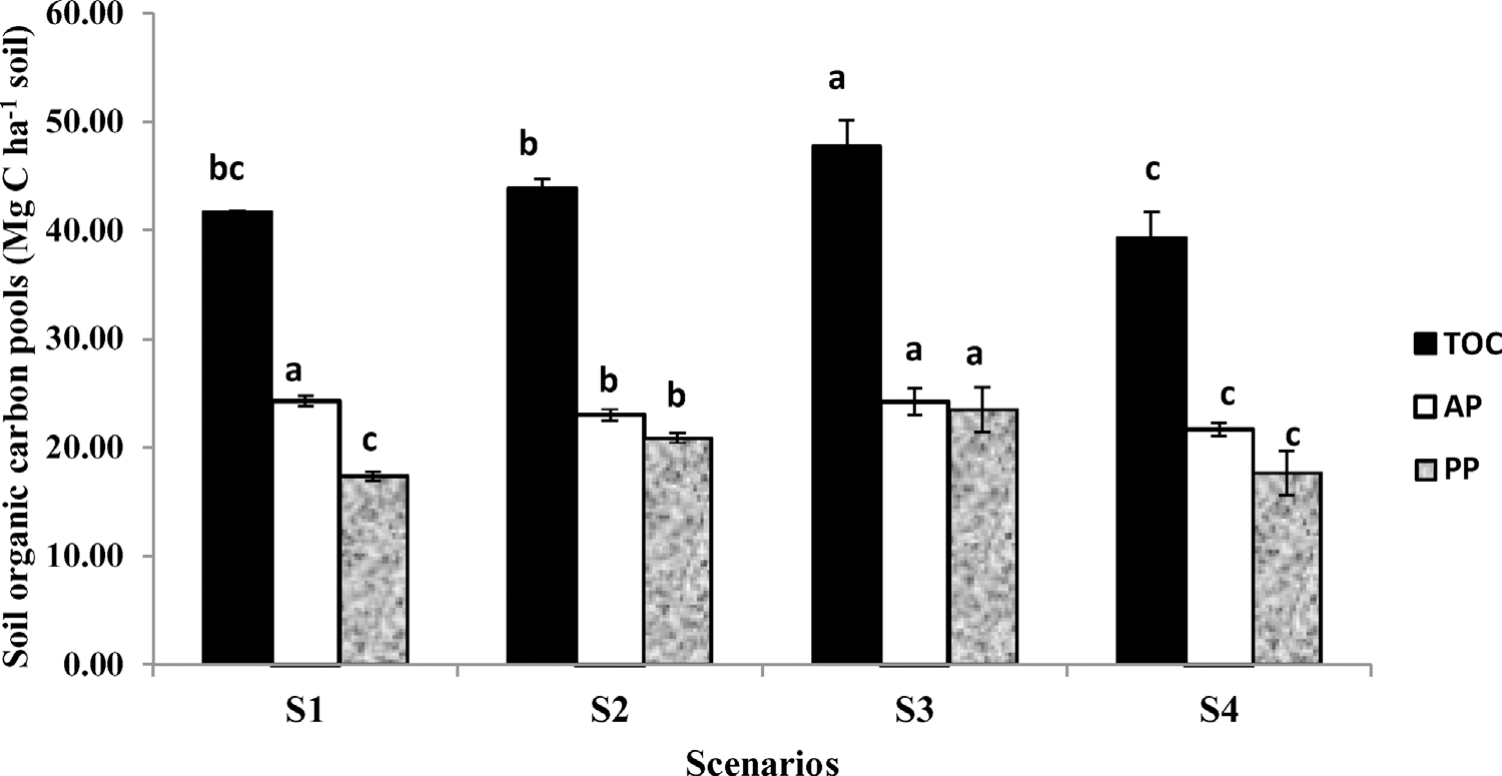
Total active (AP) and passive pool (PP) (Mg C ha^−1^ soil) of soil organic carbon in 0–30 cm depth soil as affected by different tillage and crop management practices followed under four scenarios. Different small letters denote significant difference among the values of C stock, AP and PP between the scenarios. The vertical lines above the bars represent the standard deviation. S1 (TPR-CTW): conventional till puddled transplanted rice- conventional tille wheat; S2 (TPR/MTNPR + R-ZTW + R-CTMB + R): conventional till puddled transplanted rice/machine transplanted non puddle rice with residue- zero till wheat with residue- conventional till mung bean with residue; S3 (ZTDSR + R-ZTW + R-ZTC/ZTMB + R): zero till direct seeded rice with residue-zero till wheat with residue-zero till cowpea/zero till mung bean with residue; S4 [NPTPR/ZTDSR + R-CT(P + M)/ZTM + R-ZTC/ZTM + R]: non puddle transplanted rice/zero till direct seeded rice with residue-conventional till potato and maize intercrop/zero till mustard with residue-zero till cowpea/zero till maize with residue. Scenario details are given in Table 2, Fig. 2, Fig. 3.

Apart from this, crops in all scenarios were grown according to the recommended agronomic practices (Fig. 4).

### 2.3 Soil sampling and processing

Composite soil sample (soil sample were collected from ten randomly selected points within a plot) from 0 to 10, 10–20 and 20–30 cm depth were collected in the year 2016 after harvest of winter crops with the help of soil auger. The samples were air-dried and passed through 2 mm sieve for analysis of total organic carbon (TOC) and its fractions. Sub samples of the collected soil were taken and stored in a freezer at 4 °C for analysis of biological properties (enzymatic activity) of soil.

### 2.4 Soil analysis

#### 2.4.1 Total organic carbon (TOC) and C stock

Total organic carbon of soil sample was analysed by improved chromic acid digestion method through externally heating the sample at 135° C for 30 min (Haenes, 1984). The standard Walkey and Black (1934) method is based on oxidation of organic matter by K_2_Cr_2_O_7_ with H_2_SO_4_ heat of dilution. Due to less heat of dilution the method was associated with incomplete recovery of SOC. It has been reported that the standard Walkey and Black (1934) method accounts for 60–86% recovery of total soil organic carbon as a result of incomplete oxidation (Walkey and Black, 1934). Thus, to overcome the problem of less recovery of SOC, TOC in soil was determined by improved chromic acid digestion method (Haenes, 1984).

#### 2.4.2 Organic carbon of different oxidisability

Different fractions of TOC were determined under an increasing gradient of oxidising condition using three sulphuric acid (H_2_SO_4_)-aqueous solution ratio of 0.5:1, 1:1 and 2:1 corresponding to 12, 18 and 24 N H_2_SO_4_, respectively (Chan et al., 2001). Carbon oxidized by 24 N H_2_SO_4_ equivalents to oxidisable carbon obtained by standard Walkey and Black (1934) method. The amount of carbon thus estimated leads to partition of TOC into the following four different organic carbon pools of decreasing oxidisability.

Fraction I (very labile): organic carbon oxidisable under 12.0 N H_2_SO_4_

Fraction II (labile): the difference in carbon oxidisable under 18.0 N and 12.0 N H_2_SO_4_

Fraction III (less labile): the difference in carbon oxidisable under 24.0 N and 18.0 N H_2_SO_4_

Fraction IV (non-labile): the difference between TOC and carbon oxidisable under 24.0 N H_2_SO_4_

#### 2.4.3 Active pool (AP) and passive pool (PP) of organic carbon

Active pool of organic carbon was computed by adding fraction I and fraction II, whereas, passive pool of organic carbon was determined as addition of fraction 3 and fraction 4. Active pool of organic carbon represents amount of organic carbon present in easily oxidisable form in soil. Whereas, passive pool of organic carbon is resistant to decomposition, thus, it has higher mean residence time in soil. Hence, from soil carbon sequestration point of view storing more carbon in passive pool is important.

The size of organic carbon stock for all pools of SOC was calculated by multiplying their respective SOC value with bulk density and depth of soil as:

Carbon stock in soil = SOC × Bulk density (BD) × Depth × 10

Where, SOC is expressed in g kg^−1^ soil, BD in Mg m^−3^, depth in m and C stock in Mg ha^−1^.

#### 2.4.4 Carbon budgeting

Through carbon budgeting, quantitative increase or decrease in total, AP and PP C stock of S2–S4 was calculated over S1 in 0–30 cm depth of soil through following formula (Mandal et al., 2008):

$$Carbon\;\;\;left\;\;\;in\;\;\;soil=\overset{0.3}{\underset{d=0.0}{\sum }}(C_{org}-C_{ct})$$

Similarly the percentage increase or decrease of respective C stocks (total, AP, PP) of S2–S4 over S1 was also calculated using the following expression (Mandal et al., 2007):

$$Carbon\;\;\;\;\;build\;\;\;up\;\;(\%)=\overset{0.3}{\underset{d=0.0}{\sum }}\frac{C_{org-C_{ct}}}{C_{ct}}\times 100$$

Where, C_org_ stands for total/AP/PP C stock (Mg ha^−1^ soil) of in S2/S3/S4 and C_ct_ represents total/AP/PP C stock (Mg ha^−1^ soil) in scenario 1. 0.3 stands for 30 cm depth of soil expressed in m.

The data generated through carbon budgeting provides us a clear understanding of the allocation of increase or decrease in total C stock under S2–S4 over S1 in 0–30 cm depth of soil to their respective AP and PP.

#### 2.4.5 Biological properties in soil

Microbial biomass carbon (MBC) in soil was measured by the method of Nunan et al. (1998) with some modification as mentioned in Parihara et al. (2016). Dehydrogenase activity in soil was estimated by the procedure outlined by Casida et al. (1964). Fluoresein diacetate (FDA) hydrolytic activity in soil was determined using the procedure mentioned by Green et al. (2006). Alkaline phosphatase activity was determined following the method Tabatabai and Bremner (1969).

#### 2.4.6 Crop harvest and yield estimation

At crop maturity, wheat and rice were harvested and threshed with a combine harvester in all the scenarios except S1. In S1, wheat and rice were manually harvested at ground level, and threshing was done using a plot thresher. Mustard, mung bean and maize were harvested manually. Mung bean pods were picked manually at 7-days intervals, and the plants were left in the field after harvest. Grain yields of wheat and rice and seed yield of mung bean, mustard and maize were determined by manually harvesting four areas of 5 m × 4 m in each plot from within each grid cell, giving a total harvested area of 20 m^2^ in each plot. The harvested area for yield estimation was 5 m × 6 m for mustard, maize, and mung bean in four locations in each of the four grid cells. Grain yield of wheat, maize, rice, and mung bean was reported at 12, 14, 14, and 12% grain moisture, respectively. The yields of all non-rice crops were converted to rice equivalent yield using Eq. (1) for the estimation of annual total system yield. In addition, the yields of crops (mustard and maize) in S4 were converted to wheat and mung bean equivalent yields using Eqs. (2) and (3), respectively, for comparison with S1–S3.

(1) $$Rice\;\;\;\;equivalent\;\;\;of\;\;\;non\;\;\;rice\;\;\;crop\;\;yield=\frac{Non\;\;\;rice\;\;crop\;yield\;(Mgha^{-1})\times MSP\;\;of\;\;non\;\;rice\;\;crop\left(INRkg^{-1}\right)}{MSP\;\;\;of\;\;rice\;\;crop\left(INRkg^{-1}\right)}$$

(2) $$Wheat\;\;\;equivalent\;\;\;of\;mustard\;\;\;yield=\frac{Mustard\;\;\;crop\;\;yield\;(Mg\;\;\;ha^{-1})\times MSP\;\;of\;\;mustard\;\;\left(INRkg^{-1}\right)}{MSP\;\;\;of\;\;wheat\;\;crop\;\;\left(INR\;\;\;\;kg^{-1}\right)}$$

(3) $$\text{Mungbean}\;\;\text{ equivalent}\;\;\text{ of}\;\;\text{ maize}\;\;\text{ yield}=\frac{Maize\;\;\;crop\;\;yield\;(Mg\;\;ha^{-1})\times MSP\;\;of\;\;\;maiz\left(INR\;\;\;\;\;kg^{-1}\right)}{MSP\;\;\;of\;\;\;\;mung\;\;\;bean\;\;\;crop\;\;\left(INR\;\;\;kg^{-1}\right)}$$

where MSP of cereals, oilseeds and pulses is the minimum support price in Indian rupees (INR) fixed by the Government of India. Here, we report the yield of crops for the last three years, because from that period some changes in the scenarios were made.

#### 2.4.7 Above ground crop residue biomass estimation

No above ground crop residue was incorporated/retained in S1, while S2–S4 received crop residues (Table 2). For biomass estimation of crop residue in S2–S4, 1 m^2^ area of above ground residues retained/incorporated was cut manually from three locations in net plot giving a total harvested area of 3 m^2^ in each plot.

### 2.5 Statistical analysis

The data generated were subjected to analysis of variance (ANOVA) technique to know significant difference among treatments. Duncan’s Multiple Range Test (DMRT) test was used for multiple comparisons among the treatments at *p < 0.05* using SPSS programme (ver. 16.0). Similarly, correlation analysis was carried out using the same statistical software.

## 3 Results

### 3.1 Total organic carbon stock (TOC stock)

Long term CA practice significantly influenced TOC stock in different scenarios and depths (Table 3). Scenario 3 (full CA) recorded significantly higher TOC stock (47.71 ± 2.46 Mg C ha^−1^ soil) as compared to other scenarios in the total depth of soil studied. On the contrary, S4 (diversified cropping system with high cropping intensity) showed significantly lower C stock (39.33 ± 2.40 Mg C ha^−1^) than all other scenarios. On an average, TOC stock in different scenarios follows the order: S3 (47.71 ± 2.46) > S2 (43.91 ± 0.84) > S1 (41.65 ± 0.13) > S4 (39.33 ± 2.40 Mg C ha^−1^ soil). Maximum accumulation of SOC (19.41 ± 1.84 Mg C ha^−1^) in top depth of soil was observed under S3 followed by S4 (16.56 ± 1.71 Mg C ha^−1^), S2 (16.53 ± 0.78 Mg C ha^−1^) and S1 (16.22 ± 0.60 Mg C ha^−1^) and SOC accumulation reduced in lower depths (Table 3). In 10–20 cm depth significantly low SOC was observed in S4 (12.61 ± 0.10 Mg C ha^−1^) and statistically at par values of SOC were obtained in rest scenarios (S1–S3). In 20–30 cm soil depth significantly greater SOC accumulation was recorded in S2 (12.82 ± 1.10 Mg C ha^−1^) and S3 (13.10 ± 0.21 Mg C ha^−1^) in comparison to S1 (10.36 ± 1.07 Mg C ha^−1^) and S4 (10.16 ± 0.80 Mg C ha^−1^).

**Table 3:** Depth wise (0–10, 10–20, 20–30 cm) distribution of total organic carbon stock (Mg C ha^−1^ soil) (Mean ± SD) as affected by different tillage and crop management practices followed in four scenarios.

Scenarios^1^	0–10 cm	10–20 cm	20–30 cm	Total
S1: TPR-CTW	16.22 ± 0.60^b^	15.06 ± 0.92^a^	10.36 ± 1.07^b^	41.65 ± 0.13^BC^
S2: TPR/MTNPR + R-ZTW + R-CTMB + R	16.53 ± 0.78^b^	14.56 ± 0.65^a^	12.82 ± 1.10^a^	43.91 ± 0.84^B^
S3: ZTDSR + R-ZTW + R-ZTC/ZTMB + R	19.41 ± 1.84^a^	15.20 ± 0.73^a^	13.10 ± 0.21^a^	47.71 ± 2.46^A^
S4: NPTPR/ZTDSR + R-CT(P + M)/ZTM + R-ZTC/ZTM + R	16.56 ± 1.71^b^	12.61 ± 0.10^b^	10.16 ± 0.80^b^	39.33 ± 2.40^C^

^1^For scenario details refer Table 2, Fig. 2, Fig. 3.

Different capital letters and small letters denote significant difference among the values of total and depths respectively, across the scenarios.

### 3.2 Organic carbon fractions of different degree of oxidisability

Across all the scenarios, comparatively higher proportions of different oxidisable fractions were found in top soil and decreased with depth increment except in fraction IV, where higher value was obtained in lower depths under S2 and S3 (Table 4). The magnitudes followed the order: fraction I > fraction IV > fraction II > fraction III in S1 and S4, and fraction IV > fraction I > fraction II > fraction III in S2 and S3. S3 recorded significant higher value of SOC under fraction I (16.21 ± 1.59 Mg C ha^−1^) and fraction IV (18.93 ± 2.12 Mg C ha^−1^), while, S1, S2 and S4 registered higher SOC under fraction II and fraction III. Considerable variation was also obtained in AP (active pool) and PP (passive pool) SOC under different scenarios as a result of difference in oxidisable fractions (Fig. 4). S1 and S3 recorded significantly higher SOC under AP than S2 and S4 and constitute 58.30 and 50.75% of their respective C stock. Similarly, in terms of PP SOC S2 and S3 which are significantly different (S3 being superior to S2) were statistically superior to S1 and S4 and constitute 47.63 and 49.25% of their respective C stock. The ratio of AP to PP followed the order of S1 (1.40) > S4 (1.23) > S2 (1.10) > S3 (1.03).

**Table 4:** Organic carbon fractions (Mg C ha^−1^ soil) (Mean ± SD) of different degree of oxidisability in different depths (0–10, 10–20, 20–30 cm) of soil as affected by different tillage and crop management practices followed in four scenarios.

Scenarios	Fraction I (very labile carbon)				Fraction II (labile carbon)			
	0–10 cm	10–20 cm	20–30 cm	Total	0–10 cm	10–20 cm	20–30 cm	Total
S1: TPR-CTW	5.31 ± 0.14^b^	4.30 ± 0.24^a^	4.25 ± 0.13^a^	13.86 ± 0.01^B^	4.45 ± 0.31^a^	3.33 ± 0.29^a^	2.64 ± 0.23^a^	10.42 ± 0.48^A^
S2: TPR/MTNPR + R-ZTW + R-CTMB + R	5.87A ± 0.35^b^	4.89 ± 0.40^a^	3.71 ± 0.34^a^	14.47 ± 0.36^B^	3.61 ± 0.11^ab^	2.65 ± 0.22^b^	2.26 ± 0.46^ab^	8.52 ± 0.55^B^
S3: ZTDSR + R-ZTW + R-ZTC/ZTMB + R	7.15 ± 1.07^a^	5.30 ± 0.57^a^	3.76 ± 0.57^a^	16.21 ± 1.59^A^	3.15 ± 1.18^b^	3.01 ± 0.01^ab^	1.85 ± 0.25^b^	8.01 ± 1.23^B^
S4: NPTPR/ZTDSR + R-CT(P + M)/ZTM + R-ZTC/ZTM + R	5.49 ± 0.94^b^	4.48 ± 0.71^a^	3.60 ± 0.27^a^	13.58 ± 0.20^B^	3.44 ± 0.46^ab^	2.54 ± 0.68^b^	2.11 ± 0.34^ab^	8.09 ± 0.62^B^

Scenarios	Fraction III (less labile carbon)				Fraction IV (non labile carbon)			
	0–10 cm	10–20 cm	20–30 cm	Total	0–10 cm	10–20 cm	20–30 cm	total
S1: TPR-CTW	2.41 ± 0.29^b^	2.68 ± 1.01^a^	1.35 ± 0.23^a^	6.45 ± 1.11^A^	4.05 ± 0.75^b^	4.75 ± 0.75^a^	2.12 ± 0.75^b^	10.92 ± 1.09^C^
S2: TPR/MTNPR + R-ZTW + R-CTMB + R	2.42 ± 0.44^b^	2.13 ± 0.32^a^	1.21 ± 0.17^a^	5.77 ± 0.58^A^	4.63 ± 0.04^b^	4.88 ± 0.40^a^	5.63 ± 0.83^a^	15.15 ± 0.99^B^
S3: ZTDSR + R-ZTW + R-ZTC/ZTMB + R	1.91 ± 0.29^b^	1.25 ± 0.20^a^	1.41 ± 0.11^a^	4.57 ± 0.11^A^	7.21 ± 2.47^a^	5.64 ± 0.50^a^	6.08 ± 0.61^a^	18.93 ± 2.12^A^
S4: NPTPR/ZTDSR + R-CT(P + M)/ZTM + R-ZTC/ZTM + R	3.12 ± 0.53^a^	2.21 ± 1.08^a^	1.10 ± 0.45^a^	6.42 ± 1.66^A^	4.51 ± 1.71^b^	3.38 ± 0.80^b^	3.35 ± 0.70^b^	11.24 ± 2.62^C^

^1^For scenario details refer Table 2, Fig. 2, Fig. 3.

Different capital letters and small letters denote significant difference among the values of total and depths respectively, across the scenarios.

### 3.3 Carbon budgeting

There was net build up of TOC in S2 (2.27 ± 0.73 Mg C ha^−1^) and S3 (6.07 ± 2.34 Mg C ha^−1^), which accounts for 5.44 and 14.56% increase over S1, respectively (Table 5). But opposite effect was observed in S4, where net depletion was recorded in comparison to S1 and the magnitude was −2.32 ± 0.61 Mg C ha^−1^. Further, carbon build up was also computed in AP and PP SOC. The result revealed that all scenarios (S2, S3 and S4) registered negative build up of SOC in AP and positive build up in PP. The order of magnitude was S4 (−2.61 ± 0.83 Mg C ha^−1^) > S2 (−1.28 ± 0.02 Mg C ha^−1^) > S3 (−0.06 ± 0.01 Mg C ha^−1^) for AP and S3 (6.13 ± 2.09 Mg C ha^−1^) > S2 (3.55 ± 0.75 Mg C ha^−1^) > S4 (0.30 ± 0.05 Mg C ha^−1^) for PP.

**Table 5:** Carbon left (mean ± SD) and carbon build up (%) (Mean ± SD) in soil as affected by different tillage and crop management practices followed in three scenarios (S2, S3 and S4) over S1.

Scenarios^1^	Carbon left in soil Mg C ha-1 soil			Carbon build up (%)		
	Total	AP	PP	Total	AP	PP
S2: TPR/MTNPR + R-ZTW + R-CTMB + R	2.27 ± 0.73	−1.28 ± 0.02	3.55 ± 0.75	5.44 ± 1.75	−5.28 ± 0.05	20.50 ± 4.72
S3: ZTDSR + R-ZTW + R-ZTC/ZTMB + R	6.07 ± 2.34	−0.06 ± 0.01	6.13 ± 2.09	14.56 ± 4.58	−0.23 ± 0.01	35.36 ± 7.37
S4: NPTPR/ZTDSR + R-CT(P + M)/ZTM + R-ZTC/ZTM + R	−2.32 ± 0.61	−2.61 ± 0.83	0.30 ± 0.05	−5.57 ± 1.44	−10.72 ± 2.13	1.87 ± 0.25

^1^For Scenario details refer Table 2, Fig. 2, Fig. 3.

*AP: active pool; PP: passive pool.

### 3.4 Soil enzymatic activities

The effect of different management practices on soil microbial biomass carbon (MBC) and enzymatic activities such as dehydrogenase (DHA), flouroscein diacetate (FDA) and alkaline phosphatise activity (AlkP) are given in Table 6. Gradual decrease in MBC and enzymatic activities were observed with depth increment. Significantly greater FDA was observed in S3 (49.54 ± 2.07 mg flouroscein kg^−1^ soil h^−1^) and S4 (48.12 ± 0.56 mg flouroscein kg^−1^ soil h^−1^) in 0–10 cm depth than S1 (43.51 ± 1.57 mg flouroscein kg^−1^ soil h^−1^) and S2 (44.96 ± 1.67 mg flouroscein kg^−1^ soil h^−1^). The highest MBC in 0–10 cm soil depth was recorded in S3 (89.32 ± 3.46 μg C g^−1^ soil) followed by S4 (88.17 ± 3.27 μg C g^−1^ soil), S2 (83.76 ± 0.75 μg C g^−1^ soil) and S1 (69.87 ± 2.52 μg C g^−1^ soil). S3 and S4 were found to be statistically at par and were significantly higher than S1 and S2. Value of MBC for S1 was significantly lower among all scenarios.

**Table 6:** Microbial Biomass Carbon (MBC) (mean ± SD) and enzymatic activities (mean ± SD) in different depths (0–10, 10–20, 20–30 cm) of soil as affected by different tillage and crop management practices followed in four scenarios.

Scenarios	Soil depths (cm)			
	0–10 cm	10–20 cm	20–30 cm	Mean
**Microbial Biomass Carbon (MBC) [μg C gsoil^−1^]**				
S1: TPR-CTW	69.87 ± 2.52^c^	25.99 ± 2.21^c^	23.61 ± 2.73^a^	39.82 ± 0.91^A^
S2: TPR/MTNPR + R-ZTW + R-CTMB + R	83.76 ± 0.75^b^	22.09 ± 2.17^d^	19.50 ± 3.04^a^	41.79 ± 1.35^A^
S3: ZTDSR + R-ZTW + R-ZTC/ZTMB + R	89.32 ± 3.46^a^	35.40 ± 1.51^a^	21.15 ± 3.30^a^	48.62 ± 2.38^A^
S4: NPTPR/ZTDSR + R-CT(P + M)/ZTM + R-ZTC/ZTM + R	88.17 ± 3.27^a^	30.12 ± 2.63^b^	18.64 ± 0.67^a^	45.64 ± 0.63^A^
**Dehydrogenase activity (DHA) [μg TPF h^−1^ gsoil^−1^]**				
S1: TPR-CTW	9.88 ± 0.81^a^	8.01 ± 0.19^a^	5.73 ± 0.72^a^	7.87 ± 0.37^A^
S2: TPR/MTNPR + R-ZTW + R-CTMB + R	9.03 ± 0.64^a^	8.20 ± 0.87^a^	5.72 ± 0.33^a^	7.65 ± 0.37^A^
S3: ZTDSR + R-ZTW + R-ZTC/ZTMB + R	9.34 ± 0.56^a^	7.91 ± 1.40^a^	5.60 ± 0.61^a^	7.61 ± 0.44^A^
S4: NPTPR/ZTDSR + R-CT(P + M)/ZTM + R-ZTC/ZTM + R	9.53 ± 0.27^a^	7.59 ± 0.30^a^	5.07 ± 0.30^a^	7.40 ± 0.07^A^
**Flourosceindiacetate activity (FDA) [mg flouroscein kg soil^−1^hr^−1^]**				
S1: TPR-CTW	43.51 ± 1.57^b^	42.88 ± 1.95^a^	31.24 ± 1.16^a^	39.21 ± 0.25^A^
S2: TPR/MTNPR + R-ZTW + R-CTMB + R	44.96 ± 1.67^b^	33.97 ± 2.33^b^	25.17 ± 2.33^a^	34.70 ± 1.44^A^
S3: ZTDSR + R-ZTW + R-ZTC/ZTMB + R	49.54 ± 2.07^a^	35.46 ± 1.14^b^	22.92 ± 8.50^ab^	35.97 ± 2.67^A^
S4: NPTPR/ZTDSR + R-CT(P + M)/ZTM + R-ZTC/ZTM + R	48.12 ± 0.56^a^	31.89 ± 1.72^c^	13.78 ± 1.29^b^	31.26 ± 0.80^A^
**Alkaline phosphatise activity (AlkP) [μg *p*-nitrophenol g soil^−1^** **hr^−1^]**				
S1: TPR-CTW	269.8 ± 6.16^a^	232.4 ± 21.57^a^	210.0 ± 11.88^a^	237.4 ± 5.70^A^
S2: TPR/MTNPR + R-ZTW + R-CTMB + R	285.8 ± 28.80^a^	230.9 ± 10.10^a^	184.0 ± 12.98^a^	233.6 ± 11.11^AB^
S3: ZTDSR + R-ZTW + R-ZTC/ZTMB + R	287.8 ± 46.94^a^	234.3 ± 22.70^a^	151.3 ± 17.84^b^	224.4 ± 21.73^AB^
S4: NPTPR/ZTDSR + R-CT(P + M)/ZTM + R-ZTC/ZTM + R	257.9 ± 53.07^a^	195.0 ± 22.66^b^	147.4 ± 22.65^b^	200.1 ± 14.78^C^

^1^For scenario details refer Table 2, Fig. 2, Fig. 3.

Different capital letters and small letters denote significant difference among the values of mean and depths respectively, across the scenarios.

### 3.5 Grain yields of different crops and system grain rice equivalent yield

Rice grain yield in the year 2013–14 was significantly higher in S2 (6.4 ± 0.24 Mg ha^−1^) than rest scenarios and S3 (4.8 ± 0.18 Mg ha^−1^) and S4 (4.6 ± 0.21 Mg ha^−1^) recorded significantly lower rice grain yield than both S1 (5.5 ± 0.36 Mg ha^−1^) and S2 (6.4 ± 0.24 Mg ha^−1^) (Table 7). However, in the year 2014-15 significantly higher rice grain yield was observed in S3 (7.5 ± 0.14 Mg ha^−1^) than rest scenarios. In contrast to year 2014-15, in year 2015-16, significantly lower rice grain yield was observed in S3 (5.6 ± 0.45 Mg ha^−1^) than S2 (6 ± 0.23 Mg ha^−1^) and S4 (6.2 ± 0.30 Mg ha^−1^). On the contrary, wheat grain yield was significantly enhanced under S3 in individual years and mean of years. Moreover, S2 (4.8 ± 0.18 Mg ha^−1^) showed significant higher mean wheat grain yield than S1 (4.3 ± 0.31 Mg ha^−1^) and S4 (4.5 ± 0.23 Mg ha^−1^), which were statistically at par. The mean wheat grain yield followed the order: S3 > S2 > S4 > S1. Unlike to both rice and wheat grain yields, mung bean grain yield was found significantly greater in S4 (1.8 ± 0.10 Mg ha^−1^) than all other scenarios and S3 (1.4 ± 0.05 Mg ha^−1^) produced significantly more mungbean grain than S2 (1.3 ± 0.05 Mg ha^−1^). Again, although much variability in mean grain yields of different crops were obtained in different scenarios, when system productivity in terms of rice equivalent yield was compared, S2, S3 and S4 showed statistically at par values and were significantly greater than S1and the order followed: S4 (16.39 ± 0.84 Mg ha^−1^) > S3 (16.08 ± 0.58 Mg ha^−1^) > S2 (15.72 ± 0.61 Mg ha^−1^) > S1 (10.32 ± 0.67 Mg ha^−1^).

**Table 7:** Year wise, mean of years (mean ± SD) and system grain yield (mean ± SD) as affected by different tillage and crop management practices followed in four cropping system scenarios.

Scenarios^1^	Rice yield (Mg ha^−1^)				Wheat yield (Mg ha^−1^)				Mung bean yield (Mg ha^−1^)				System rice equivalent yield (Mg ha^−1^)			
	2013−14	2014−15	2015−16	Mean	2013−14	2014−15	2015−16	Mean	2013−14	2014−15	2015−16	Mean	2013−14	2014−15	2015−16	Mean
S1	5.5 ± 0.36^b^	6.7 ± 0.35^b^	5.3 ± 0.34^b^	5.8 ± 0.35^B^	4.1 ± 0.25^c^	4.7 ± 0.36^b^	4.2 ± 0.33^c^	4.3 ± 0.31^C^	–	–	–	–	9.7 ± 0.62^c^	11.5 ± 0.72^c^	9.6 ± 0.67^c^	10.3 ± 0.67^B^
S2	6.4 ± 0.24^a^	6.6 ± 0.25^b^	6.0 ± 0.23^a^	6.3 ± 0.24^AB^	4.4 ± 0.17^b^	5.2 ± 0.20^a^	4.9 ± 0.18^ab^	4.8 ± 0.18^B^	1.5 ± 0.06^c^	1.6 ± 0.06^a^	0.7 ± 0.05^b^	1.3 ± 0.05^C^	16.1 ± 0.61^b^	17.4 ± 0.66^b^	13.6 ± 0.57^b^	15.7 ± 0.61^A^
S3	4.8 ± 0.18^c^	7.5 ± 0.14^a^	5.6 ± 0.45^b^	6.0 ± 0.25^B^	5.1 ± 0.17^a^	5.2 ± 0.16^a^	5.1 ± 0.17^a^	5.1 ± 0.17^AB^	2.0 ± 0.06^a^	1.6 ± 0.05^a^	0.6 ± 0.03^c^	1.4 ± 0.05^B^	16.9 ± 0.56^a^	18.3 ± 0.48^a^	12.9 ± 0.72^b^	16.1 ± 0.58^A^
S4	4.6 ± 0.21^c^	6.2 ± 0.25^c^	6.2 ± 0.30^a^	5.7 ± 0.25^B^	4.2 ± 0.18^bc*^	4.8 ± 0.22^b*^	4.6 ± 0.30^b*^	4.5 ± 0.23^C*^	1.9 ± 0.09^b#^	1.7 ± 0.10^a#^	1.7 ± 0.13^a#^	1.8 ± 0.10^A#^	15.4 ± 0.69^b^	17.0 ± 0.79^b^	16.7 ± 1.04^a^	16.4 ± 0.84^A^

^1^For scenario details refer Table 2, Fig. 2, Fig. 3.

Different capital letters and small letters denote significant difference among the values of mean and individual years respectively, across the scenarios.

*Wheat equivalent yield of mustard.

#Mungbean equivalent yield of maize.

## 4 Discussion

### 4.1 Total and depth wise variation of organic carbon stock

Maximum increase in TOC stock under S3 might be due to the highest addition of crop residues coupled with conservation tillage (Ghosh et al., 2012, Das et al., 2013). Ploughing of soil causes breakage of macro-aggregates into micro-aggregate and silt and clay size particles inside soil (Beare et al., 1994, Bronick and Lal, 2005) exposing protected organic carbon inside macro-aggregate for oxidation (Six et al., 2000). In S2 and S3 summer fallow period was utilised through cultivation of leguminous crop, which resulted into augmentation of SOC. This finding was in line with the result reported by (Ghosh et al., 2012). Past studies also revealed that fallowing lessens SOC through reduction in recycling of non-harvested crop residue into the soil (Calegari et al., 2008) and SOC is enhanced by increasing cropping intensity (Hutchinson et al., 2007) by adding more crop biomass in soil. Conservation tillage with residue incorporation slows down the decomposition rate of added residue increasing organic carbon concentration in soil (Dick, 1983). In the present set of experiment also stabilization of organic carbon in soil was found to enhance with increasing amount of crop residue added (Table 8) and adoption of higher level of CA practice (Table 2). Thus, lower TOC stock was observed in S1 in comparison to S2 and S3, where CA and crop residue retention was practised. The lowest TOC stock value was recorded in S4, which shows the effect of cropping system and quality of crop residue added. Adoption of partial CA and following highly nutrient exhaustive cropping system (Maize and Potato intercropping) in initial 4 years of experiment under S4 might deplete organic carbon from soil and the effect counterbalanced the positive impact of CA and crop residue addition. In addition to this, increasing crop diversity in S4 increased diversity of carbon substrate though litter fall, this in turn could increased microbial biomass, microbial diversity and decomposition rate (Bardgett and Shine, 1999, Gartner and Cardon, 2005) leading to net deletion of SOC in the scenario. Higher microbial activity could also result into break down of aggregates with concomitant depletion of SOC.

**Table 8:** Annual above ground crop residue (mean ± SD) retained after grain harvest of preceding crop (Mg ha^−1^) in four scenarios.

Scenario^1^	2013−14				2014−15				Average
	Winter (rice)*	Summer (wheat)	Rainy (mungbean/maize)	Total	Winter (rice)	Summer (wheat)	Rainy (mungbean/maize)	Total	
S1: TPR-CTW	0	0	0	0	0	0	0	0	0
S2: TPR/MTNPR + R-ZTW + R-CTMB + R	2.86 ± 0.27	2.45 ± 0.25	1.45 ± 0.22	6.76 ± 0.71	2.65 ± 0.32	2.35 ± 0.29	1.41 ± 0.26	6.41 ± 0.75	6.58 ± 1.05
S3: ZTDSR + R-ZTW + R-ZTC/ZTMB + R	3.33 ± 0.38	2.65 ± 0.27	1.72 ± 0.23	7.70 ± 0.93	3.00 ± 0.56	2.60 ± 0.34	1.37 ± 0.27	6.97 ± 0.81	7.33 ± 1.13
S4: NPTPR/ZTDSR + R-CT(P + M)/ZTM + R-ZTC/ZTM + R	2.5 ± 0.26	0.91 ± 0.19#	2.87 ± 0.30**	6.28 ± 0.72	2.80 ± 0.35	0.81 ± 0.24#	3.14 ± 0.51**	6.75 ± 1.1	6.51 ± 1.02

^1^For scenario details refer Table 2, Fig. 2, Fig. 3.

*Name in parenthesis is of the previous crop residue.

*In rainy season, residues refer to maize stubble.

# In summer, residue refers to winter mustard in place of wheat.

The principal cause of higher enrichment of SOC on top depth was more crop residue addition on top soil in comparison to soil of lower depth. Along with this, the root growth is limited by lesser nutrient and microbial activity in lower depth resulting in lower total addition of crop residues in lower depth (Ingram and Fernandes, 2001; Sharma et al., 1992; Tiwari et al., 1995). SOC declined along the depths in all scenarios but it varied significantly among the scenarios (Table 3). The reason for variation in depth distribution of SOC in different scenarios could be attributed to higher root biomass addition in lower depth through legume crop under S2 and S3 (mungbean) (Ganeshamurthy, 2009). Additionally, legumes in cropping system leads to increase in amount of water soluble carbon in soil (Mazzarino et al., 1993, Campbell et al., 1999) and some dissolved organic matter (DOM), which is released though decomposition of crop residues inform of soluble intermediate into the soil solution may be translocated to lower depth (Guggenberger et al., 1994) increasing organic carbon in 20–30 cm soil under S2 and S3. In contrast to S2 and S3, negative build up of carbon over S1 was recorded under S4 in 10–20 cm; this may be due to the cumulative effect of elimination leguminous crop from last three cropping cycle, of adopting nutrient exhaustive cropping system for initial 4 years and of higher decomposition of SOC by addition of diverse litters. Analysis of C stock values in 0–30 cm depth of soil and its depth distribution across the scenarios explicitly explained the fact that CA practice and crop intensification augment SOC status in general as in S2 and S3, but it fails to do so where there is increase in cropping intensity with nutrient exhaustive crops and without legume crops as in S4. Thus, it can be concluded that management practices followed under S3 was best among other scenarios for enhancing SOC and for better soil health. These evidences fulfilled part of objective of our study.

### 4.2 Variation in different carbon fractions, pools and microbial parameters

Higher proportion of different oxidisable fractions in top soil was due to higher microbial activity arising from addition of mineralizable organic matter in form of crop residues (Kaur et al., 2008, Naik et al., 2017). To this an exceptionally higher SOC observed under fraction IV (non labile carbon) in lower depths of S2 and S3 was possibly due to rapid conversion of crop residue biomass and labile carbon fractions (mung bean root and DOM) to recalcitrant form, and its persistence under favourable condition of moisture and minimal soil disturbance (Sreekanth et al., 2013). Besides this, the added OC in lower depth got chemically stabilized though silt and clay fractions of soil in form of more stable carbon (Lutzow et al., 2006), resulting in significant higher total carbon in fraction IV under S2 and S3. The aforesaid causes served the major reasons for getting two different order of magnitude of carbon under four different fractions across the scenarios. Higher SOC content was recorded in fraction II and fraction III under S1, S2 and S4 in comparison to S3. This could be due to conversion of labile carbon to more resistant fraction under anaerobic condition prevailing in transplanted rice in S1, S2 and S4. This finding is in agreement with Ghosh et al. (2012), who reported higher SOC in fraction 3 under transplanted rice condition. Significantly higher AP SOC recorded under S1 and S3 due their significantly higher labile carbon content, but under different fractions. Higher labile carbon content in S1 and S3 were due to their significantly higher SOC content in fraction II and fraction I, respectively. Similarly, PP SOC was recorded significantly higher in S2 and S3 due to their significantly higher SOC in fraction IV. Lowest AP SOC to PP SOC ratio in S3 suggests that S3 is superior in carbon sequestration and maintaining soil quality.

Microbial parameters (MBC, FDA) were found to be increased with increase in residue carbon addition. Fresh residues supplied readily mineralisable and hydrolisable carbon for better microbial growth. With depth increment decline in all microbial parameters studied were due to decrease in supply of carbon input. This section emphasized that management practices followed in S3 was superior to that of other scenarios in increasing both labile and non labile carbon in soil. Thus, S3 was best from soil health and carbon sequestration point of view. It provided some insight to distribution of SOC in different pools, thus fulfilled the SOC stabilization part of our objective.

### 4.3 Carbon budgeting

Positive values of total and PP SOC and negative values of AP SOC in carbon left in soil and carbon build up percentage under S2 and S3 demonstrated that there was increase in allocation of PP SOC and decease in allocation of AP SOC of total increase in SOC over S1. This suggested that the management practices followed in S2 and S3 promoted more SOC in PP and less SOC in AP after seven years of CA and crop intensification practice. However, very low negative value of AP SOC under S3 is negligible and increase in allocation of PP SOC sufficiently higher (1.73 times) than S2, thus, it can be concluded that S3 was the best management practice in enhancing both labile and non-labile carbon in soil. Positive value of PP SOC and negative value of AP SOC in soil under S3 does not mean that there was no increase in SOC in AP under S3, because here all data were calculated in reference to S1. In fact, S3 maintained statistically at par value of AP SOC with S1 and was statistically superior to other scenarios in terms of PP SOC (Fig. 4). Carbon budgeting analysis gave more insight to carbon stabilization in different scenarios as affected by different cropping systems and management practices and provides evidence for carbon stabilization part of our objective.

### 4.4 Variation in yield crops in different scenarios

Significantly higher rice grain yield in S3 in year 2014–15 than all other scenarios could be due to many benefits derived out of CA practices namely, improved soil moisture retention, better aggregation and bulk density and ultimately due to enhanced SOC content with better nutrient recycling in S3 having ZT-DSR with residue retention. In addition to this rice grain yield was significantly greater in S1 and S2 than S4 in the same year, indicating that puddling provides better micro environment like anaerobic condition, less weed competition and less percolation for more growth and productivity of rice. Significantly lower rice grain yield in years 2013–14 and 2015–16 under S3 were due to yield reduction as a result of high rainfall and mealy bug (*Psedococcidae* spp.), respectively. Direct seeded rice (DSR) generally matures one week before transplanted rice and root system of transplanted rice hold soil more firmly than that of DSR. In year 2013-14, during 40th and 41st standard meteorological week there was occurrence of heavy rainfall along with wind. At the same time, DSR in S3 and S4 was in milking stage, while transplanted rice in S2 was in panicle initiation stage. This caused DSR heavier than transplanted rice; consequently, the rainstorm caused lodging of DSR crop in S3 and S4 resulting in yield reduction. In year 2015-16, there was higher proliferation of *Brachiaria* spp. (a grassy weed, which acts as alternate as well as collateral host to Mealy bug) in S3, because, in DSR generally higher proliferation of weed is observed in comparison to transplanted rice. Consequently, partial yield loss in S3 was observed as a result infestation of Mealy bug. However, in S4, where DSR was also adopted higher proliferation of the above mentioned weed was not there. This may be due to diversified cropping system adopted in S4. In fact, weed seed bank dynamics in soil depends upon the previous crop and management practices followed in the particular system. Hence, the cropping system and management practices followed in S4 might not have allowed that particular weed in the DSR crop. The mean rice grain yield did not show any significant difference among the scenarios. Improvement in labile carbon fraction, C stock and better soil physical condition were some favourable factors created by CA practice responsible for the significant increase in wheat grain yield in S2 and S3 in comparison to S1 and S4. Jat et al. (2014) also reported higher rice-wheat system grain yield under ZT-DSR and ZT-wheat in comparison to CT-rice and CT-wheat in eastern IGP. Significantly higher mung bean equivalent yield in S4 was due to higher maize minimum support price in comparison to mung bean. Besides, statistically at par values in system grain yield observed under S2–S4 was due to higher grain yield obtained in separate crops in each scenario. Lowest system yield in S1 was due to poor soil and crop management practices. This section fulfilled our crop yield evaluation part of our objective of the study.

Moreover, Laik et al. (2014) working on same set of experiment reported higher water productivity in S2–S4 in comparison to S1 and followed the order: S4 > S3 > S2 > S1. In addition to this they also reported the highest benefit cost ratio in S3 due to the lowest cost of crop cultivation associated to it.

## 5 Conclusion

The present experiment established that CA practice enhanced carbon stock upto 0–30 cm depth of soil, which is evident from increase in carbon stock in S2 and S3 in comparison to S1 (farmers practice). In this study, with increase in cropping intensity carbon stock was enhanced under S2 and S3 but declined in S4. This result emphasizes that carbon stock in soil not only depend upon amount of crop residues returned to soil but also depend on quality of crop residue, which varies according to crop selected in a particular cropping system. The present CA based experiment caused an increase in TOC stock in lower depths (20–30 cm) with stabilization of SOC in passive pool compared to small or negligible increment in SOC in some past studies. Inclusion of pulses in the cropping systems caused an enhancement of SOC in lower soil depths and identified as sink of OC, hence, increasing cropping intensity with legume crop is highly recommended. The S3 scenario proved to be highly efficient in terms of enhancing carbon sequestration and labile carbon in the system. Although, S4 performed well in terms of system grain yield, but it was not sustainable due to low SOC content. Hence, cropping system and management practice under scenario 3 was adjudged as the best among the four scenarios in maintaining higher carbon build up and stabilization resulting in better soil health and food security. However, in the future studies some alternative diversified cropping system should be evaluated in Indo-Gangetic Plain of South Asia, which will be profitable to farmer as well as sustainable from environment, soil health and food security.
